# Net clinical benefit of patiromer for acute hyperkalemia: a post-hoc analysis of the reduce trial

**DOI:** 10.1080/07853890.2025.2581912

**Published:** 2025-11-03

**Authors:** Robert McArthur, W. Frank Peacock, Jeff Budden, Zubaid Rafique

**Affiliations:** ^a^ Department of Emergency Medicine, Baylor College of Medicine, Houston, TX, USA; ^b^ Vifor Pharma Group, Redwood City, CA, USA

**Keywords:** Hyperkalemia, potassium, electrolyte, pharmacology

## Abstract

**Introduction:**

Hyperkalemia (HK) is a common and potentially life-threatening electrolyte disturbance managed with potassium (K)-shifting medications and potassium binders. The use of K-shifting agents is a confounder when assessing the effects of K-binding treatments in clinical trials. Patiromer is a K-binding agent with demonstrated efficacy for the management of chronic hyperkalemia. As both changes in sK level and the number of interventions required to lower potassium are clinically relevant, we propose an evaluation using Net Clinical Benefit (NCB), defined as the number of K shifting interventions less the numerical change in K.

**Methods:**

We conducted a post-hoc analysis of the REDUCE trial to determine the NCB of patients with hyperkalemia in the ED setting. Patients were randomized to the standard of care (SOC) or patiromer plus standard of care (PAT). Serum potassium and HK-related interventions were recorded. NCB, defined as the number of K shifting interventions less the numerical change in K, with lower numbers showing a greater benefit, was calculated at 2, 4, and 6 h for the standard of care (SOC) and patiromer (PAT) groups.

**Results:**

Of the 43 patients randomized, 30 completed 4 h of study intervention and were included in the analysis, including 15 each in the SOC and PAT arms. NCB for PAT over SOC was numerically superior at 2 h (−0.05 vs 0.43; *p* = 0.108), 4 h (0.11 vs 0.73; *p* = 0.097), and 6 h (0.64 vs 1.60; *p* = 0.094) post-intervention.

**Discussion:**

NCB captures the clinical benefit of binders, while accounting for the confounding effect of other potassium-lowering agents.

## Introduction

Hyperkalemia (HK) is a common and potentially life-threatening electrolyte disturbance, characterized by serum potassium (sK) levels above 5.5 mEq/L. It accounts for more than 800,000 ED visits or 2-3% of annual ED visits in the United States [1–3]. Hyperkalemia most commonly occurs due to decreased potassium excretion secondary to chronic kidney disease. Additionally, hyperkalemia may result from increased release of intracellular potassium due to crush injuries, burns or rhabdomyolysis [3]. In cases of severe hyperkalemia (usually sK > 6.5 mEq/L), the loss of K gradient across cardiac myocyte membranes alters depolarization and may lead to ventricular arrhythmias and sudden cardiac death [4].

The management of hyperkalemia rarely involves a single intervention. Rather, success is a function of titration, where therapy requires multiple significant interventions, each with a risk of adverse events. Standard therapies for potassium shifting include insulin, which has been shown to induce hypoglycemia in up to 75% of patients and albuterol which commonly induces tachycardia [5,6]. Therefore, evaluating target achievement alone is insufficient, as interventions requiring multiple therapies or dose adjustments may predispose patients to higher risks of adverse events. We derived Net Clinical Benefit (NCB) as a simple difference between change in sK and number of interventions required to achieve that sK to promote ease of calculation and because both values were of similar scale in the REDUCE trial. By accounting for both the change in sK and the number of exposures to potassium shifting agents, we propose that NCB more accurately captures the desired effect of potassium binder treatment and should be considered when evaluating titratable therapies in the emergency department.

Patiromer is a K-binding agent that is FDA approved and has demonstrated efficacy in the management of chronic hyperkalemia across multiple large randomized controlled trials [7–11]. Data for emergency department use of patiromer is limited. The REDUCE trial, a pilot study on use of patiromer in the ED, showed that patiromer lowered sK concentration at 2 h post-administration, but this effect was not sustained at 6 h [12]. Prior work evaluating the efficacy of potassium binders in the acute setting has primarily focused on lowering sK; however, this outcome may not capture the full effect of potassium binder therapy. Given the temporary nature of potassium lowering with insulin and albuterol, most patients require multiple treatments for HK management in the ED [13]. Hence the ideal treatment option would be to achieve normokalemia with the least number of additional interventions. As both changes in sK level and the number of interventions required to lower potassium levels are clinically relevant, we propose an evaluation of patiromer using NCB. The REDUCE trial was a previously published open-label prospective randomized standard therapy-controlled evaluation of patients for the treatment of hyperkalemia. Thus, this study aims to determine the net clinical benefit of Patiromer for acute hyperkalemia through a post hoc analysis of the REDUCE trial.

## Patients and methods

We conducted a post-hoc analysis of the REDUCE trial as a pilot study to determine the NCB of patiromer for management of hyperkalemia in the ED setting. The inclusion criteria were patients aged ≥ 18 years with a history of end-stage renal disease and an sK≥ 6.0mEq/L. Patients were excluded for significant arrhythmia on initial ECG, known allergy to patiromer, treatment with a potassium binder other than patiromer, or pregnancy. Patients were randomized to the standard of care (SOC) or a single dose of 25.2 g patiromer plus standard of care (PAT). The treating providers were blinded to the treatment assignment, and SOC interventions were administered as per the treatment provider. Serum potassium and HK-related interventions were recorded at baseline and at 1, 2, 4, 6, and 8 h after enrollment. This was a post-hoc analysis of de-identified data from the REDUCE trial. The REDUCE trial was approved by the Baylor College of Medicine Institutional Review Board (protocol H-39340), was registered at ClinicalTrials.gov (NCT02607085) and study participants provided written informed consent for inclusion. This secondary analysis of deidentified data was exempt from IRB review.

NCB, defined as the number of K shifting interventions less than the numerical change in K, with lower numbers showing a greater benefit, was calculated at 2, 4, and 6 h for the SOC and PAT groups. Means were compared using an independent t-test. Statistical analyses were performed using Microsoft Excel (Microsoft Corporation. Redmond, Washington).

NCB=Number  of  K  shifti ng  interventions−change  in serum  K

## Results

Of the 43 patients randomized, 30 completed 4 h of study intervention and were included in the analysis, including 15 each in the SOC and PAT arms. The treatment arms of PAT vs. SOC had no significant difference in age (41 vs. 48; *p* = 0.35), sex (66.7% male vs. 46.7% male), or baseline potassium measurement (6.4 vs 6.7; *p* = 0.129).

Mean change in sK was larger for PAT vs SOC at 2 h (−0.52 vs −0.04, *p* = 0.04), 4 h (−0.69 vs −0.60, *p* = 0.37) and 6 h (−0.61 vs −0.36, *p* = 0.31) yet only achieved significance at 2 h. Mean number of potassium shifting agents utilized for PAT vs SOC was equal at 2 h (0.6 vs 0.6, *p* = 0.50), but numerically lower at 4 h (0.8 vs 1.33, *p* = 0.10) and 6 h (1.11 vs 1.90, *p* = 0.11) while not achieving a significant difference at any time point.

**Figure 1.**
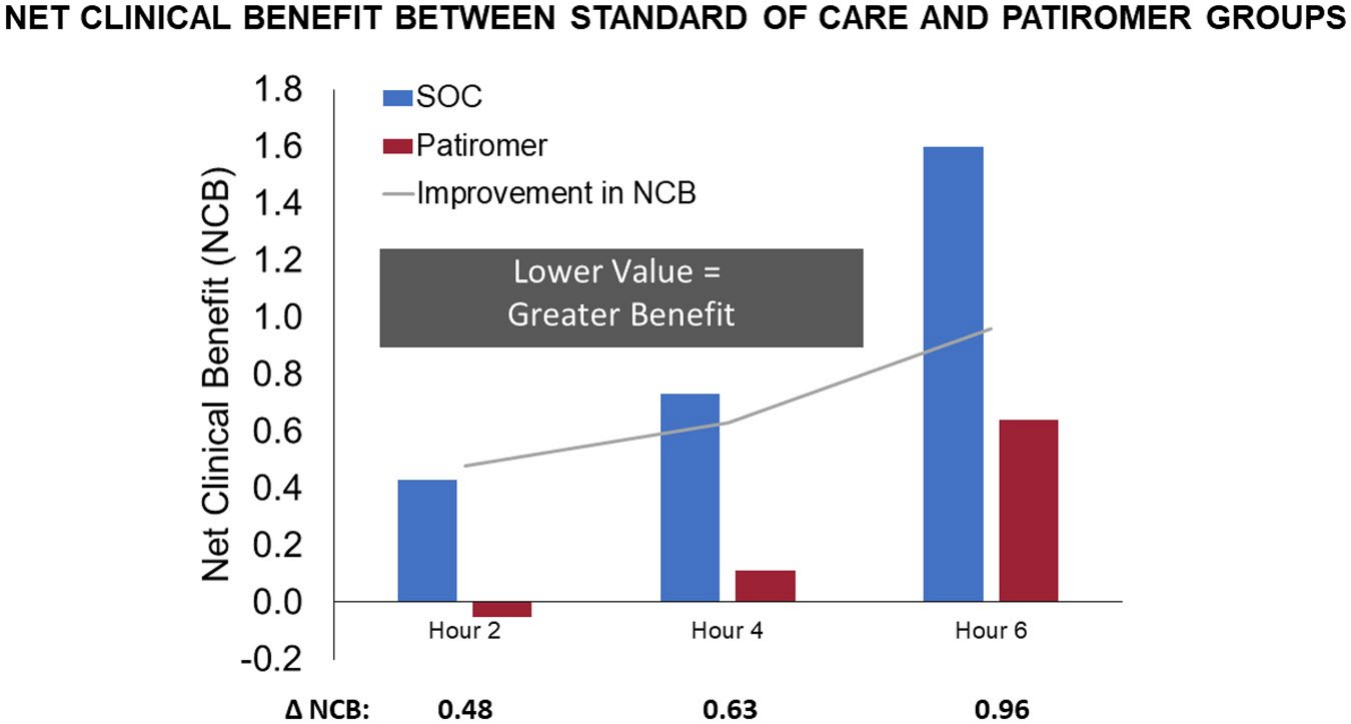
Net clinical benefit of SOC vs PAT over time. ΔNCB = Net Clinical Benefit

NCB for PAT over SOC was numerically superior at 2 h (−0.05 vs 0.43; *p* = 0.108), 4 h (0.11 vs 0.73; *p* = 0.097), and 6 h (0.64 vs 1.60; *p* = 0.094) post-intervention. While the effect of PAT on NCB did not reach statistical significance in this small pilot study, there was consistent numerical superiority, with a trend of improved NCB and increasing statistical significance with time (Figure 1).

## Discussion

Potassium-shifting agents transiently but reliably lower sK, thus making them ethically necessary for treating acute HK in clinical trials. However, their use increases the risk of adverse events (AEs). Following administration of insulin for HK, hypoglycemia may occur in up to 75% of patients, and tachycardia is common following albuterol administration [5,6]. Given the risk of adverse events associated with K-shifting agents, it is imperative that this impact be considered when evaluating potential therapies for HK. Additionally, the efficacy of K-shifting agents in lowering sK is highly variable, which introduces a significant confounder in the analysis of K-binding agent efficacy. In unblinded trials and observational studies, patients receiving patiromer may be administered fewer additional potassium-lowering interventions compared to the control group, which could diminish the apparent potassium-lowering effectiveness of patiromer and render the study results difficult to interpret.

While extensive prior work has demonstrated the efficacy of patiromer for management of chronic hyperkalemia [7–11]. Similar data for acute hyperkalemia in the ED setting is lacking. One retrospective study by Goriacko et al. reported patiromer provided no added benefit over insulin monotherapy for HK highlighting the need for further work to characterize the roll of patiromer for management of acute HK [14].

In the REDUCE trial, patients treated with a single dose of patiromer were noted to have a significant decrease in sK compared to SOC at 2 h. This effect was not sustained at the 4 and 6 h marks however the SOC group required more potassium shifting treatments to achieve this equivalent sK. In our study NCB was numerically greater at 2, 4 and 6 h, yet did not achieve statistical significance in this small sample size. By addressing both change in sK and number of interventions required to achieve it, NCB captures the clinical benefit of binders, while accounting for the risk of adverse events and the confounding effect of other potassium-lowering agents.

The present work has several limitations, as a pilot study the present study suffers from a small sample size. Furthermore, data were obtained from secondary analysis of a prior trial.

## Conclusion

Given the need to account for the confounding effects of potassium shifting agents, NCB may be a superior measure for the evaluation of potassium binders. Our post-hoc analysis supports the use of NCB in future work on potassium binders for acute management of hyperkalemia. Future work with a larger sample size is necessary to ensure the generalizability of the present work.

## Data Availability

The data that support the findings of this study are available from the corresponding author, RM, upon reasonable request.
